# Propranolol potentiates the anti-angiogenic effects and anti-tumor efficacy of chemotherapy agents: implication in breast cancer treatment

**DOI:** 10.18632/oncotarget.343

**Published:** 2011-10-17

**Authors:** Eddy Pasquier, Joseph Ciccolini, Manon Carre, Sarah Giacometti, Raphaelle Fanciullino, Charlotte Pouchy, Marie-Pierre Montero, Cindy Serdjebi, Maria Kavallaris, Nicolas André

**Affiliations:** ^1^Children's Cancer Institute Australia, Lowy Cancer Research Centre, UNSW, Randwick, NSW, Australia; ^2^Pharmacokinetics Unit, UMR-MD3, Aix-Marseille Univ, Marseille, France; ^3^INSERM UMR 911, Centre de Recherche en Oncologie biologique et en Oncopharmacologie, Aix-Marseille Univ, Marseille; ^4^Hematology & Pediatric Oncology Department, La Timone University Hospital of Marseille, France; ^5^Metronomics Global Health Initiative, Marseille, France; ^†^Université Pierre et Marie Curie - Univ Paris 6, CNRS UMR 7211, INSERM U959, Paris, France

**Keywords:** breast cancer, angiogenesis, propranolol, beta-adrenergic receptor antagonist, chemotherapy, combination therapy

## Abstract

Recent clinical evidence revealed that the use of beta-blockers such as propranolol, prior to diagnosis or concurrently with chemotherapy, could increase relapse-free and overall survival in breast cancer patients. We therefore hypothesized that propranolol may be able to increase the efficacy of chemotherapy either through direct effects on cancer cells or *via* anti-angiogenic mechanisms. *In vitro* proliferation assay showed that propranolol (from 50-100 μM) induces dose-dependent anti-proliferative effects in a panel of 9 human cancer and “normal” cell lines. Matrigel assays revealed that propranolol displays potent anti-angiogenic properties at non-toxic concentrations (<50 μM) but exert no vascular-disrupting activity. Combining chemotherapeutic drugs, such as 5-fluorouracil (5-FU) or paclitaxel, with propranolol at the lowest effective concentration resulted in synergistic, additive or antagonistic effects on cell proliferation *in vitro* depending on the cell type and the dose of chemotherapy used. Interestingly, breast cancer and vascular endothelial cells were among the most responsive to these combinations. Furthermore, Matrigel assays indicated that low concentrations of propranolol (10 – 50 μM) potentiated the anti-angiogenic effects of 5-FU and paclitaxel. Using an orthotopic xenograft model of triple-negative breast cancer, based on injection of luciferase-expressing MDA-MB-231 cells in the mammary fat pad of nude mice, we showed that propranolol, when used alone, induced only transient anti-tumor effects, if at all, and did not increase median survival. However, the combination of propranolol with chemotherapy resulted in more profound and sustained anti-tumor effects and significantly increased the survival benefits induced by chemotherapy alone (+19% and +79% in median survival for the combination as compared with 5-FU alone and paclitaxel alone, respectively; p<0.05). Collectively our results show that propranolol can potentiate the anti-angiogenic effects and anti-tumor efficacy of chemotherapy. The current study, together with retrospective clinical data, strongly suggests that the use of propranolol concurrently with chemotherapy may improve the outcome of breast cancer patients, thus providing a strong rationale for the evaluation of this drug combination in prospective clinical studies.

## INTRODUCTION

The costs associated with drug development are rising so dramatically that cheaper, faster and more effective strategies to identify new compounds and implement them successfully in the clinic are desperately needed [1]. In the current financial and economical context, drug repositioning, which consists of using already approved drugs for new medical indications, is gaining increasing interest as an alternative strategy for drug development [2-4]. By using drugs with well-known toxicity and pharmacokinetics profiles, this strategy can remove several years, substantial risks and costs from the pathway to the clinic. For instance, the recent discovery of the efficacy of propranolol -a non-selective β-adrenergic receptor (β-AR) antagonist commonly used for the treatment of hypertension- in treating severe infantile haemangioma has revolutionized the management of this pathology in 2 years [5, 6]. Although the first evidence for a role of β-ARs in cancer progression was provided more than 20 years ago [7], the serendipitous observation of the efficacy of propranolol in treating haemangiomas has renewed interest in exploring the anti-angiogenic and anti-cancer properties of β-AR antagonists (also called β-blockers).

A number of *in vitro* studies have demonstrated the anti-proliferative, anti-migratory and cytotoxic properties of propranolol, particularly against lung adenocarcinoma [7, 8], colon carcinoma [9], breast carcinoma [10], nasopharyngeal carcinoma [11], ovarian cancer [12], pancreatic cancer [13-15] and gastric cancer cells [16]. Propranolol was also found to exert potent anti-angiogenic effects *in vitro* through direct mechanisms on vascular endothelial cells [17, 18] and by decreasing pro-angiogenic signaling in both stromal [19] and cancer cells [11, 20-23]. Some of these promising anti-cancer properties have been confirmed *in vivo* using different animal models of human cancers. Propranolol was thus found to exert potent cancer preventive effects in models of chemically-induced lung and pancreatic cancers [24, 25]. Furthermore, innovative pre-clinical models of breast and ovarian cancer showed that propranolol was able to specifically inhibit stress-induced tumor growth and metastatic spread through anti-angiogenic and immuno-stimulatory mechanisms [26, 27].

Over the past 12 months, emerging clinical data have further strengthened the potential benefits of β-AR antagonists in cancer patients [28, 29]. Three retrospective clinical studies revealed that the use of β-AR antagonists, prior to diagnosis and/or concomitantly with chemotherapy, was associated with increased survival and/or decreased metastatic spread and incidence of tumor recurrence in breast cancer patients [30-32]. Different molecular mechanisms have been proposed to explain such benefits of β-AR antagonists in breast cancer patients [28, 29], focusing on the anti-metastatic properties of β-AR antagonists alone. Here, we hypothesized for the first time that β-AR antagonists, and propranolol in particular, may be able to increase the efficacy of chemotherapy by potentiating the anti-proliferative and/or anti-angiogenic effects of chemotherapeutic drugs. We thus proposed to investigate the combination of propranolol and chemotherapy on cell proliferation and angiogenesis *in vitro* and on tumor growth inhibition and survival *in vivo* using an orthotopic model of triple-negative breast cancer.

**Figure 1:**
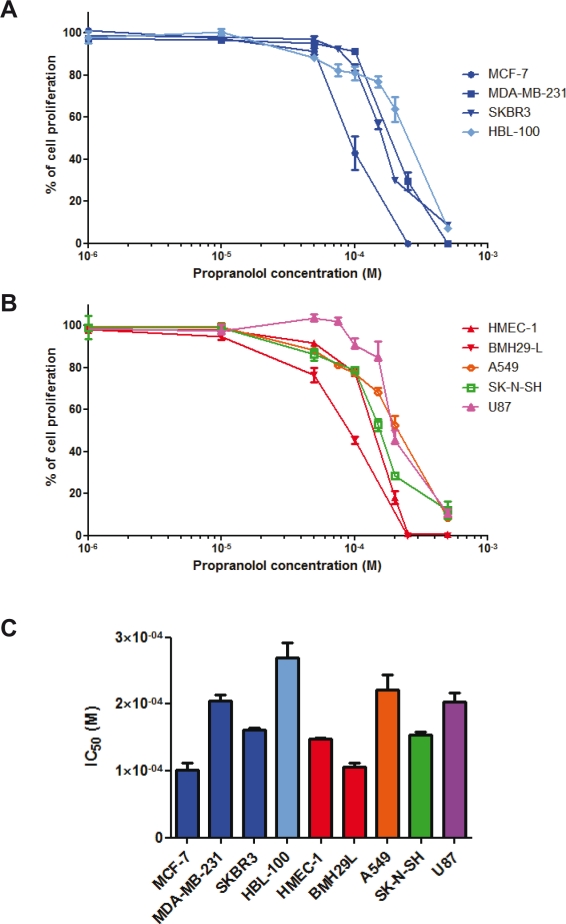
*In vitro* anti-proliferative properties of propranolol Growth inhibition assay performed on a panel of human cell lines using Alamar Blue or MTT after 72 h incubation with a range of concentrations of propranolol. Three breast adenocarcinoma cell lines (MCF-7, MDA-MB-231 and SKBR3) and a breast epithelial cell line (HBL-100) were first used (A) and then a neuroblastoma (SK-N-SH), a non-small cell lung carcinoma (A549), a glioblastoma (U87) and two vascular endothelial cell lines (HMEC-1 and BMH29L) were used (B). *Points*, % of cell proliferation as compared to untreated control cells, means of at least three individual experiments; *bars*, SE; log scale for x axis. C) Histogram representation of the molar concentration of propranolol required to inhibit 50% of cell proliferation after 72h drug incubation (IC_50_) for all tested cell lines. *Columns*, means of at least three individual experiments; *bars*, SE.

## RESULTS

### Propranolol exerts dose-dependent anti-proliferative and anti-angiogenic effects *in vitro*

To investigate the anti-proliferative properties of propranolol *in vitro* a range of human cell lines were used and comprised of 6 cancer cell lines originating from breast carcinomas (MCF-7, MDA-MB-231 and SKBR3), non-small cell lung carcinoma (A549), neuroblastoma (SK-N-SH) and glioblastoma (U87), as well as 3 “normal” cell lines including 1 breast epithelial cell line (HBL-100) and 2 vascular endothelial cell lines (HMEC-1 and BMH29L). As shown in Figure 1A & B, propranolol exerts dose-dependent anti-proliferative effects against all tested cell lines. There was however a significant variability in sensitivity to propranolol across the different cell lines with IC_50_ values ranging from 100 to 269 μM (Figure 1C). The two most sensitive cell lines were breast carcinoma cell line MCF-7 and bone marrow-derived endothelial cell line BMH29L with IC_50_ values of 100 ± 11 μM and 106 ± 5 μM, respectively. In contrast, breast epithelial cell line HBL-100 and non-small cell lung carcinoma cell line A549 were the most resistant to propranolol with IC_50_ values of 269 ± 22 μM and 222 ± 21 μM, respectively.

**Figure 2:**
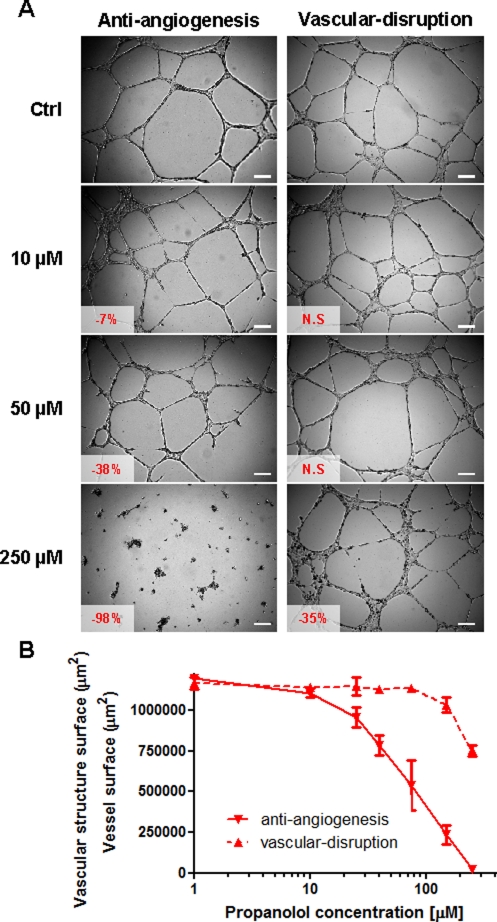
*In vitro* anti-angiogenic and vascular-disrupting properties of propranolol A) Representative photographs of BMH29L cells in Matrigel™ assays. For the anti-angiogenesis assay (*left panel*), cells were treated with a range of propranolol concentrations 20 min after seeding on Matrigel™ and photographs were taken after 8h drug incubation. For the vascular-disruption assay (*right panel*), cells were first allowed to form capillary-like structures for 6 h before drug treatment was initiated and photographs were taken after 2h drug incubation. Vascular structures were imaged on a Zeiss Axiovert 200M using a 5 X objective. *Inset*, % of inhibition/disruption as compared to untreated control cells (N.S = not statistically significant);* Scale bar*, 200 μm. B) Dose-dependent anti-angiogenic (*solid line*) and vascular-disrupting (*broken line*) effects of propranolol on BMH29L cells. *Points*, average surface occupied by vascular structures in each view field, means of at least four individual experiments; *bars*, SE; log scale for x axis.

Two types of Matrigel™ assays were used to examine both the anti-angiogenic and vascular-disrupting properties of propranolol *in vitro*. In BMH29L cells, propranolol was found to exert potent dose-dependent anti-angiogenic effects but have almost no vascular-disrupting activity (Figure 2). The formation of vascular structures was significantly inhibited after 8h incubation with low concentrations of propranolol (7, 20 and 38% inhibition at 10, 25 and 50 μM, respectively; p<0.01) compared to control, and complete angiogenesis suppression was observed at high concentrations. In contrast, propranolol concentrations of up to 150 μM induced no significant disruption of pre-formed vascular structures after 2h drug incubation and high concentrations were required to start disrupting the vascular network (35% disruption at 250 μM; p<0.001). Similar results were obtained with HMEC-1 cells (data not shown) thus confirming that propranolol is a potent anti-angiogenic but not vascular-disrupting agent.

### Propranolol potentiates the anti-proliferative effects of chemotherapy in a cell type-specific and dose-dependent manner

**Figure 3:**
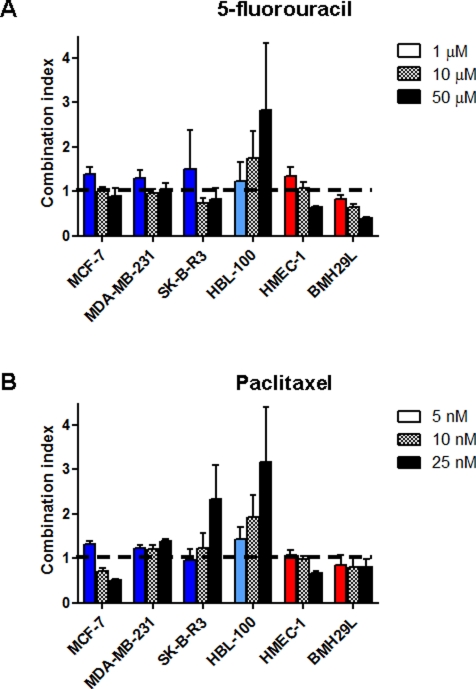
*In vitro* drug combination study of propranolol and chemotherapy on cell proliferation Histogram representation of the combination index (CI) of propranolol in association with 5-fluorouracil (A) or paclitaxel (B) on a panel of human cell lines. Growth inhibition assays were performed using Alamar Blue or MTT after 72 h incubation with a range of chemotherapeutic drug concentrations in presence or absence of propranolol at the lowest effective concentration (IC_15_). CI values were determined based on the Chou and Talalay method for all tested concentrations of chemotherapeutic drug. *Columns*, means of at least three individual experiments; *bars*, SE.

Drug combination studies using growth inhibition assay were performed to determine whether propranolol can potentiate the anti-proliferative effects of chemotherapeutic drugs. The lowest effective concentration of propranolol (IC_10_-IC_15_) was combined with a range of concentrations of the pyrimidine analog 5-fluorouracil (5-FU) and the microtubule-stabilizing agent paclitaxel in the panel of 9 human cell lines. After 72h drug incubation, cell proliferation was measured by Alamar Blue or MTT and combination index (CI) values were calculated for all tested drug concentrations based on the Chou and Talalay method [33, 34]. The CI theorem provides quantitative definition for additive effects (CI = 1), synergisms (CI<1) and antagonisms (CI>1) in drug combinations. The combination of propranolol with 5-FU resulted in a synergistic, additive or antagonistic interaction depending on the dose of 5-FU used and the cell type (Figure 3A and Table 1). In all 3 breast cancer cell lines and HMEC-1 endothelial cells, this drug combination resulted in a slight antagonism at 1 μM 5-FU but the interaction became additive or synergistic with increasing concentrations of 5-FU. In BMH29L endothelial cells, the interaction between propranolol and 5-FU was synergistic at all tested concentrations. In contrast, an antagonistic effect was observed at all tested concentrations in HBL-100 breast epithelial cells. Interestingly, in other types of cancer cell lines (*i.e.* A549, SK-N-SH and U87), the combination of propranolol and 5-FU mostly resulted in an antagonism (Table 1).

**Table 1:** Combination index of propranolol and chemotherapeutic drugs in vitro Growth inhibition assays were performed on a panel of 9 human cell lines using Alamar Blue or MTT after 72 h incubation with a range of chemotherapeutic drug concentrations in presence or absence of propranolol at the lowest effective concentration (IC_15_). Combination index values (± SE) were determined based on the Chou and Talalay method for all tested concentrations of chemotherapeutic drug. A color code is used to illustrate the different drug interactions: synergism (*green*), additivity (*yellow*), sub-additivity (*orange*) and antagonism (*red*).

	5-Fluorouracil			Paclitaxel		
Cell lines	1 μM	10 μM	50 μM	5 nM	10 nM	25 nM
MCF-7	1.38± 0.16	1.01 ± 0.08	0.89 ± 0.18	1.31 ± 0.07	0.71 ± 0.07	0.5 ± 0.03
MDA-MB-231	1.3 ± 0.18	0.96 ± 0.08	1.05 ± 0.13	1.23 ± 0.08	1.21 ± 0.1	1.38 ± 0.05
SKBR3	1.51 ± 0.87	0.74 ± 0.09	0.82 ± 0.25	0.95 ± 0.26	1.23 ± 0.34	2.32 ± 0.77
HBL-100	1.22 ± 0.44	1.76 ± 0.59	2.82 ± 1.51	1.44 ± 0.26	1.93 ± 0.51	3.18 ± 1.23
HMEC-1	1.34 ± 0.21	1.06 ± 0.14	0.64 ± 0.04	1.08 ± 0.1	0.98 ± 0.07	0.67 ± 0.05
BMH29L	0.83 ± 0.08	0.64 ± 0.08	0.40 ± 0.03	0.85 ± 0.21	0.79 ± 0.2	0.81 ± 0.17
A549	0.92 ± 0.32	1.56 ± 0.47	1.93 ± 0.61	0.73 ± 0.04	1.12 ± 0.41	1.5 ± 0.63
SK-N-SH	1.10 ± 0.43	1.39 ± 0.05	4.58 ± 1.7	0.52 ± 0.06	0.71 ± 0.08	0.70 ± 0.08
U87	6.57 ± 3.39	8.18 ± 2.02	6.6 ± 2.21	3.67 ± 1.31	2.45 ± 0.68	1.69 ± 0.91

Cell type-specific and dose-dependent effects were also observed when combining propranolol and paclitaxel (Figure 3B and Table 1). In contrast with the previous drug combination, diverse effects were observed in the 3 different breast cancer cell lines. In MCF-7 cells, the combination of propranolol and paclitaxel resulted in a slight antagonism at 1 nM paclitaxel but it became highly synergistic with increasing paclitaxel concentrations. In contrast, this drug combination led to a sub-additive effect at all tested concentrations in MDA-MB-231 cells and changed from additivity to antagonism with increasing paclitaxel concentrations in SKBR3 cells. In BMH29L cells, the combination of propranolol with paclitaxel led to a synergistic effect irrespectively of the dose of paclitaxel used whereas in HMEC-1 cells, this combination was sub-additive, additive and synergistic at 5, 10 and 25nM paclitaxel, respectively. Finally, mixed results were also obtained with the other cancer cell lines (Table 1). In A549, the interaction between paclitaxel and propranolol changed from synergism to antagonism with increasing paclitaxel concentration whereas this drug combination was synergistic and antagonistic at all tested drug concentrations in SK-N-SH and U87 cells, respectively. Propranolol thus appeared to modulate the anti-proliferative effects of chemotherapy *in vitro* in a cell type-specific and dose-dependent manner.

**Figure 4:**
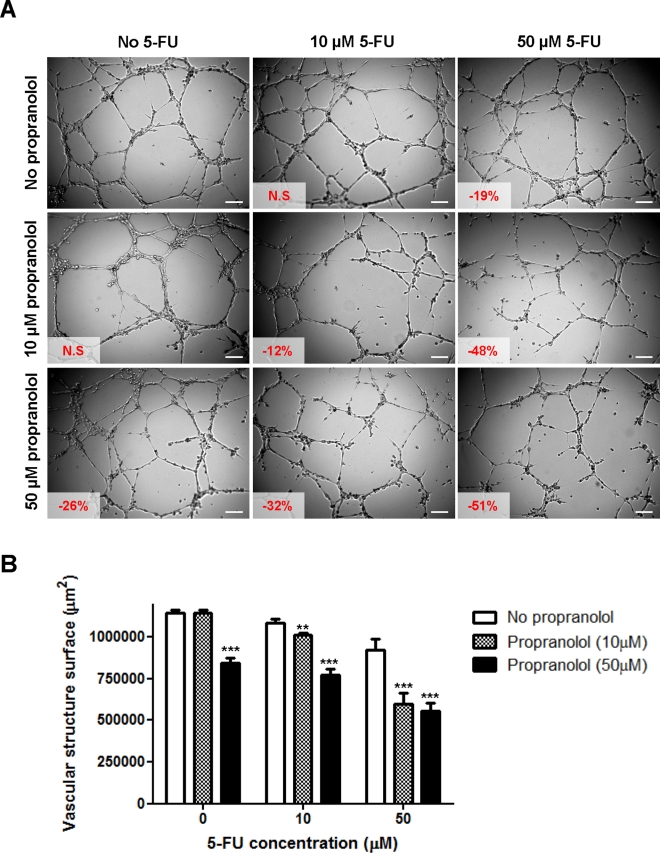
*In vitro* drug combination study of propranolol and 5-fluorouracil on angiogenesis A) Representative photographs of HMEC-1 cells in Matrigel™ assay. Cells were treated 20 min after seeding on Matrigel™ with a range of 5-fluorouracil concentrations (0, *left panel*; 10 μM, *middle panel* and 50 μM, *right panel*) in absence (*top panel*) or presence of propranolol (10 μM, *middle panel* and 50 μM, *bottom panel*) and photographs were taken after 8h drug incubation. Vascular structures were imaged on a Zeiss Axiovert 200M using a 5 X objective. *Inset*, % of inhibition as compared to untreated control cells (N.S = not statistically significant); *Scale bar*, 200 μm. B) Histogram representation of the anti-angiogenic effects of 5-fluorouracil in combination with propranolol. *Columns*, means of at least four individual experiments; *bars*, SE. Statistical analysis by ANOVA, combination vs 5-FU alone: ** p<0.01; *** p<0.001.

### Propranolol potentiates the anti-angiogenic effects of chemotherapy in vitro

To further explore the potential of propranolol to enhance the anti-cancer effects of chemotherapy, drug combination studies using Matrigel™ assay were performed. As shown in Figure 4, propranolol was able to potentiate the anti-angiogenic effects induced by 5-FU. While incubation with 10 μM of either drug alone did not significantly affect the capacity of HMEC-1 cells to form vascular structures, the combination of both drugs at this concentration resulted in a small but significant inhibition of angiogenesis (-12%; p<0.01) (Figure 4A). The presence of 10 μM propranolol further increased the inhibition of angiogenesis induced by 50 μM 5-FU from 19 to 48% (p<0.001). The effect however did not dramatically increase when higher concentrations of propranolol were used.

**Figure 5:**
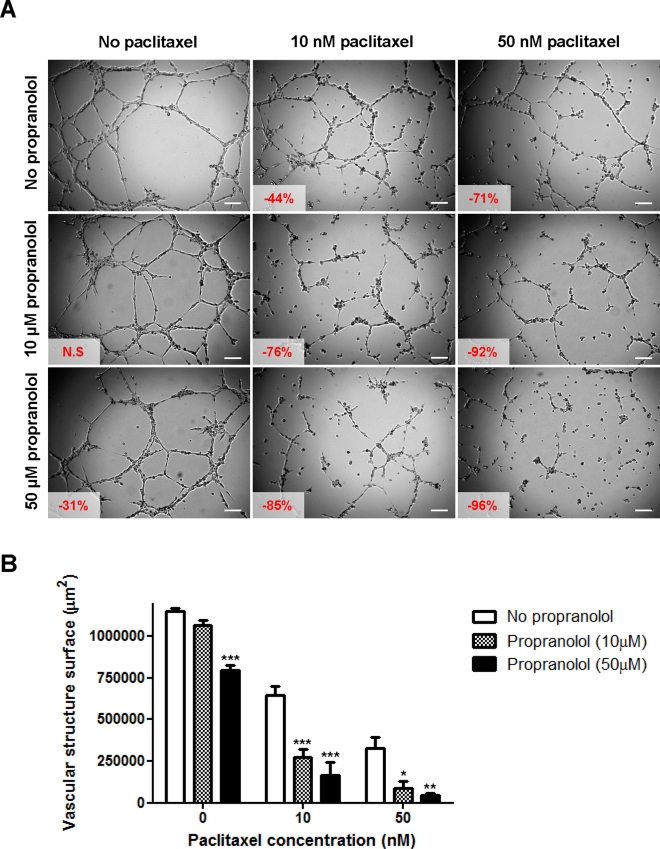
*In vitro* drug combination study of propranolol and paclitaxel on angiogenesis A) Representative photographs of HMEC-1 cells in Matrigel™ assay. Cells were treated 20 min after seeding on Matrigel™ with a range of paclitaxel concentrations (0, *left panel*; 10 nM, *middle panel* and 50 nM, *right panel*) in absence (*top panel*) or presence of propranolol (10 μM, *middle panel* and 50 μM, *bottom panel*) and photographs were taken after 8h drug incubation. Vascular structures were imaged on a Zeiss Axiovert 200M using a 5 X objective. *Inset*, % of inhibition as compared to untreated control cells (N.S = not statistically significant); *Scale bar*, 200 μm. B) Histogram representation of the anti-angiogenic effects of paclitaxel in combination with propranolol. *Columns*, means of at least four individual experiments; *bars*, SE. Statistical analysis by ANOVA, combination vs paclitaxel alone: * p<0.05; ** p<0.01; *** p<0.001.

The combination of propranolol and paclitaxel appeared to be even more potent at inhibiting angiogenesis (Figure 5). In the absence of propranolol, 10 and 50 nM paclitaxel inhibited vascular structure formation by 44 and 71%, respectively whereas in the presence of 10 μM propranolol this increased to 76 and 92% inhibition, respectively (p<0.05). Similar results were obtained with BMH29L cells (data not shown). Time-lapse videomicroscopy experiments further confirmed the increase in the anti-angiogenic effects of paclitaxel when used in combination with propranolol (supplementary videos 1-4). As compared to treatment with either drug alone, treatment with the combination resulted in incomplete morphological differentiation of endothelial cells into vascular structures, followed by regression of the few structures formed (supplementary video 4). Collectively these results demonstrate that propranolol can significantly potentiate the anti-angiogenic effects of chemotherapeutic drugs *in vitro*.

### Propranolol increases the efficacy of chemotherapy in a mouse orthotopic model of human triple-negative breast cancer

**Figure 6:**
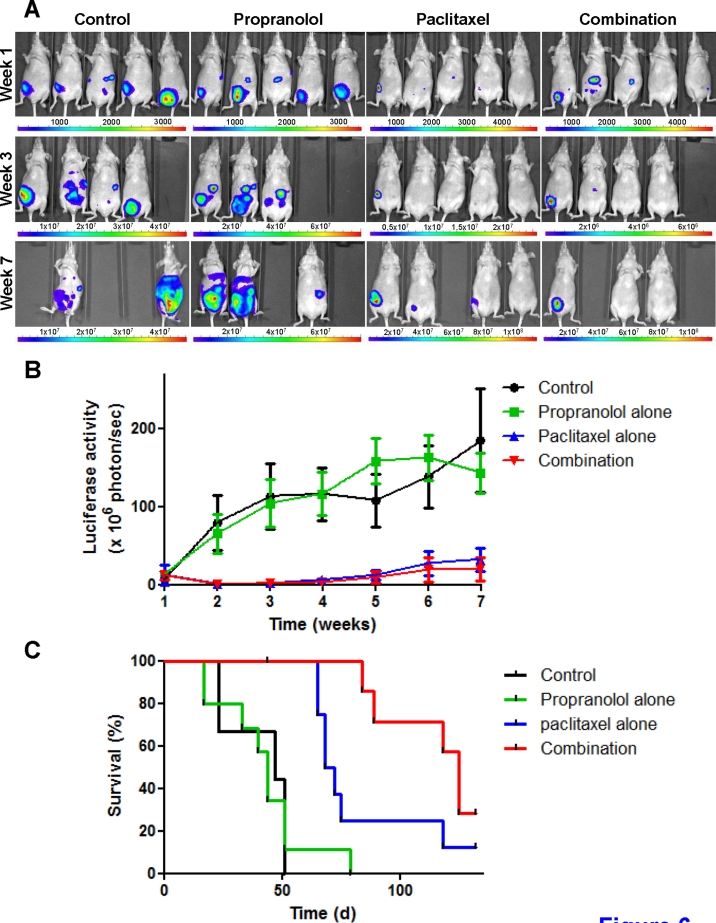
*In vitro* drug combination study of propranolol and paclitaxel NMRI nude mice were injected with luciferase-expressing MDA-MB-231 cells into the mammary fat pad and randomized into 4 groups following confirmation of engraftment. Treatment was initiated 3 weeks after tumor cell injection and consisted of i.p injection of saline 5 days a week for up to 5 weeks, 10 mg/kg propranolol alone 5 days a week for 5 weeks, 20 mg/kg paclitaxel alone 3 days a week for 3 weeks or the combination of propranolol and paclitaxel. A) Representative pictures of bioluminescence measurements performed 1 week (*top panel*), 3 weeks (*middle panel*) and 7 weeks (*bottom panel*) after treatment was initiated. The color scale indicates the signal intensity, which is directly proportional to the number of tumor cells. Pictures taken at 6 different wavelengths (i.e.,580 to 630 nm) were next combined for 3D reconstruction and tumor size evaluation. B) Quantitative analysis of tumor growth by weekly measurements of luciferase activity in mice treated with saline only (*black*; n=6), propranolol alone (*green*; n=10), paclitaxel alone (*blue*; n=8) and the combination of propranolol and paclitaxel (*red*; n=8). *Points*, average luciferase activity; *bars*, SE; time is expressed in weeks since start of treatment. C) Kaplan-Meier survival curve; time is expressed in days since start of treatment.

To investigate the effect of combining propranolol with chemotherapy on tumor growth and survival *in vivo*, a mouse orthotopic model of human triple-negative breast cancer was used and the combination of propranolol and paclitaxel was evaluated. Treatment consisted of i.p injections of paclitaxel alone (20 mg/kg, 3 days a week for 3 weeks), propranolol alone (10 mg/kg, 5 days a week for 5 weeks) or the combination of both drugs. All three treatments were well tolerated as evidenced by an absence of weight loss or change in animal well-being as compared with vehicle-only treated mice (data not shown). Weekly bioluminescence measurements showed that propranolol alone did not significantly delay tumor growth, whereas treatment with paclitaxel alone or in combination with propranolol considerably inhibited tumor progression (Figure 6A-B). The addition of propranolol to paclitaxel did not significantly increase the very potent anti-tumor effects induced by paclitaxel alone (Figure 6A-B and supplementary Figure 5). It did however induce significant survival benefits as compared to paclitaxel alone (Figure 6C and Table 2). Median survival was thus increased from 70 days in animals treated with paclitaxel alone to 125 days in animals receiving the combination of propranolol and paclitaxel. Furthermore, at study completion (150 days after start of treatment), 12.5% (1/8) and 25% (2/8) of mice were tumor-free in the cohorts of animals receiving paclitaxel alone and the combination of propranolol and paclitaxel, respectively.

Similar results were obtained when combining propranolol and 5-FU *in vivo* (supplementary Figure 6 and Table 2). The addition of propranolol to 5-FU did not significantly increase the anti-tumor effects induced by 5-FU alone, except at week 4 of treatment (p<0.05). However, this combination appeared to be the most effective treatment regimen in terms of survival benefits. Indeed, it led to a substantial increase in median survival from 44 days for both vehicle-treated and propranolol-treated mice and 47 days for mice treated with 5-FU alone to 56 days for mice treated with the combination. Our results thus demonstrate that propranolol can enhance the efficacy of chemotherapy in a mouse orthotopic model of human triple-negative breast cancer.

## DISCUSSION

Accumulating clinical evidence suggests that beta-adrenergic receptor antagonists and propranolol in particular may lead to increased survival and decreased recurrence in breast cancer patients [30-32]. Here, we provide the first evidence that propranolol can increase the efficacy of chemotherapy, either through direct effects on tumor angiogenesis and/or by potentiating the anti-angiogenic and anti-tumor effects of chemotherapeutic drugs.

Our study first demonstrated that propranolol has relatively modest anti-proliferative properties *in vitro* against a panel of 9 human cell lines. High concentrations of propranolol were required to significantly inhibit cell proliferation, with IC_50_ values ranging from 100 μM to >250 μM across the panel of cell lines. Early pharmacokinetics studies revealed that clinically relevant concentrations of propranolol are in the low μM range [35, 36], thus suggesting that propranolol alone is not likely to inhibit cell proliferation *in vivo*. The sensitivity to propranolol was cell type specific but did not depend on the tissue of origin, since all 4 cell lines of breast tissue origin displayed different levels of sensitivity. Interestingly, MCF-7 breast cancer cells were twice as much sensitive to propranolol than MDA-MB-231 cells, although the former have been reported to express low levels of β-ARs as compared to the latter [37]. This suggests that the level of β-AR expression may not directly influence cell sensitivity to the anti-proliferative effect of propranolol. In contrast with its modest effects on cell proliferation, propranolol was able to significantly inhibit angiogenesis *in vitro* at relatively low concentrations (from 10 and 20 μM in BMH29L and HMEC-1 cells, respectively). These results were consistent with those previously reported with other types of vascular endothelial cells (i.e. HUVEC and HMVEC) [18] and confirmed the potent anti-angiogenic activity of propranolol. The current study further showed that unlike microtubule-depolymerizing agents [38], propranolol has almost no vascular-disrupting activity in addition to its anti-angiogenic properties. This result indicates that the anti-vascular properties of propranolol reported in various pre-clinical and clinical studies [5, 26, 27] are mostly due to the inhibition of new capillary tube formation rather than the disruption of pre-existing vessels.

Our *in vitro* combination studies revealed that low concentrations of propranolol can modulate the anti-proliferative effects of chemotherapeutic drugs *in vitro* in a cell type-specific and dose-dependent manner. Interestingly, human breast carcinoma and vascular endothelial cell lines were among the most responsive to the combination of propranolol and chemotherapy. Consistent with our results, a recent study has shown that the combination of radiotherapy with propranolol in gastric cancer cells *in vitro* led to an increase in radiotherapy-induced apoptosis [39]. Elsewhere, β-AR agonists such as epinephrine have been previously reported to induce resistance to paclitaxel in MCF-7 breast cancer cells [40] and to 5-FU in HT-29 colon cancer cells [41] through up-regulation of the *ABCB1* gene. However, these two studies also showed that propranolol was unable to prevent epinephrine-induced overexpression of *ABCB1* and its product P-glycoprotein. This not only suggests that the potentiation of the anti-proliferative effects of 5-FU and paclitaxel by propranolol reported in the current study is independent from *ABCB1* up-regulation but also reveals that additional mechanisms, yet to be discovered, are involved in the regulation of drug sensitivity by β-AR signaling. Based on the high sensitivity of vascular endothelial cells to propranolol, we hypothesized that propranolol may also potentiate the anti-angiogenic effects of chemotherapeutic drugs. Our finding that combining low concentrations of propranolol with 5-FU or paclitaxel significantly increases angiogenesis inhibition *in vitro*, demonstrates for the first time that propranolol can potentiate the anti-angiogenic properties of chemotherapeutic drugs through direct effects on endothelial cells. Additional experiments are currently underway to unravel the mechanisms involved in the synergism between propranolol and chemotherapeutic drugs against cell proliferation and angiogenesis *in vitro*. A number of candidates are being considered. These include cAMP-protein kinase A (PKA) and Src kinase signaling pathways, which are both involved in the adrenergic regulation of cancer cell proliferation and invasion, as well as the inhibition of VEGF receptor-2 tyrosine phosphorylation and MMP-9 secretion, which have been shown to mediate the anti-angiogenic effects of propranolol *in vitro* [17, 18, 42, 43].

**Table 2:** Median survival of breast tumor-bearing mice Median survival and log-rank test values as determined from the Kaplan-Meier survival curves. Time is expressed in days since start of treatment.

	Propranolol and Paclitaxel	Propranolol and 5-Fluorouracil
Median survival (d)	Log rank test	Median survival (d)	Log rank test
Control	47	-	44	-
Propranolol	44	0.916	44	0.18
Chemotherapy	70	0.0003	47	0.026
Combination	125	0.0005	56	0.0005

Triple-negative breast cancer is estimated to affect more than 170,000 women worldwide each year and associated with a poor prognosis [44]. Effective therapeutic options are very limited but β-AR antagonists have been recently found to be particularly beneficial in triple-negative breast cancer patients [32]. The potentiation of the anti-proliferative and anti-angiogenic effects of chemotherapeutic drugs *in vitro* demonstrated in the current study provides two potential mechanisms by which propranolol may be able to increase the anti-tumor efficacy of chemotherapy *in vivo*. Previous studies have found that propranolol could inhibit the development of stress-induced metastasis in preclinical models of both ovarian and breast cancer but it only affected primary tumor growth in the ovarian cancer model [26, 27]. Here we found that propranolol alone induced only minor and transient anti-tumor effects in a mouse orthotopic model of breast cancer and it did not lead to survival benefits. This result, together with those previously reported in the literature, suggest that the anti-tumor effects of propranolol are most likely to occur in small disseminated tumors but not in larger, bulky tumors, which is consistent with the potent anti-angiogenic but weak vascular-disrupting properties of propranolol reported here. Nevertheless, our study further showed that, in absence of chronic stress, propranolol can enhance the efficacy of chemotherapy *in vivo*. Although the difference did not reach statistical significance, there was a clear trend towards a more potent anti-tumor effect when propranolol was combined with chemotherapy, as compared with chemotherapy alone. Furthermore, combining propranolol to chemotherapy resulted in improved survival in two independent *in vivo* studies. The addition of propranolol thus increased the median survival by 79% and 19% as compared to paclitaxel alone and 5-FU alone, respectively.

Collectively, our results indicate that propranolol could potentially be used concomitantly with chemotherapy in breast cancer patients to increase treatment efficacy. The evaluation of such combination therapy in prospective clinical trials is urgently needed, particularly in triple-negative breast cancer patients for whom effective therapeutic options are extremely limited. If successful, this clinical translation could rapidly impact on the treatment of more than 1 million women worldwide affected by breast cancer each year, thus further illustrating the numerous advantages of drug repositioning in terms of time- and cost-saving. Finally, it is also important to note that β-AR antagonists could also be beneficial in other types of drug-refractory solid tumors and warrants further investigation in clinically-relevant models of both adult and childhood cancers.

## MATERIAL AND METHODS

### Reagents

Propranolol (Sigma-Aldrich, Castle Hill, Australia) was resuspended in water and stock solutions (50 mM) were stored at 4°C. Commercial solutions of 5-fluorouracil in saline were obtained from La Timone University Hospital of Marseille, France and Hospira Australia (Mulgrave North, Australia) and stored at room temperature. Paclitaxel (Sigma-Aldrich) was resuspended in DMSO and stock solutions (2 mM) were stored at -20°C.

### Cell culture

The breast cancer cell line MCF-7 and glioblastoma cell line U87 were maintained in DMEM medium (Invitrogen, Mount Waverley, Australia), the breast cancer cell line MDA-MB-231, non-small cell lung cancer cell line A549 and neuroblastoma cell line SK-N-SH were maintained in RPMI-1640 medium (Invitrogen), the breast cancer cell line SKBR3 was maintained in McCoy’5A medium (Invitrogen) and the breast epithelial cell line HBL-100 was maintained in MEM (Invitrogen). All these culture media were supplemented with 10% Fetal Calf Serum (FCS), 2 mM L-glutamine and 1% penicillin streptomycin. The microvascular endothelial cell line HMEC-1 was grown on 0.1% gelatin-coated flasks using MCDB-131 medium (Invitrogen) containing 10% FCS, 2 mM L-glutamine, 1% penicillin and streptomycin, 1 μg/mL hydrocortisone and 10 ng/mL epithelial growth factor (BioScientific, Gymea, Australia). BMH29L cells are bone marrow derived endothelial cells that were immortalized by ectopic expression of human telomerase reverse transcriptase [45]. They were kindly provided by Dr Karen MacKenzie (Children's Cancer Institute Australia) and grown on 0.1% gelatin-coated flasks using Medium 199 (Invitrogen) containing 20% FCS, 5% AB-only male human serum (Sigma-Aldrich), 1% penicillin and streptomycin, 1% heparin, 5 ng/mL recombinant human FGF_β_ (fibroblast growth factor β; Sigma-Aldrich) and 20 μg/mL Endothelial Cell Growth Factor (Roche, Dee Why, Australia). All cell lines were routinely maintained in culture at 37°C and 5% CO_2_ and regularly screened to ensure the absence of mycoplasma contamination.

### Growth inhibition assay

Growth inhibition assays were performed using either Alamar blue (for MCF-7, MDA-MB-231, HMEC-1 and BMH29L cells) or MTT (for SKBR3, HBL-100, A549, SK-N-SH and U87), as previously described [46, 47]. For all cell lines, the number of cells seeded (from 1,500 to 10,000 cells per well, depending on the cell line) in 96-well plates was first optimized to ensure sustained exponential growth for 4-6 days. For all cell lines (with the exception of SK-N-SH cells, which were allowed to adhere for 72h before treatment was initiated), cells were treated 24h after seeding with a range of concentrations of i) propranolol alone (1 × 10^-7^ to 1 × 10^-4^ M), ii) chemotherapeutic drugs alone (1 × 10^-8^ to 5 × 10^-4^ M and 1 × 10^-11^ to 1 × 10^-7^ M for 5-FU and paclitaxel, respectively) or iii) chemotherapeutic drugs in combination with propranolol at the lowest effective concentration (IC_15_). After 72h drug incubation, metabolic activity was detected by addition of Alamar blue or MTT followed by spectrophotometric analysis. Cell proliferation was determined and expressed as a percentage of untreated control cells. The determination of IC_50_ values was performed using GraphPad Prism 4 software (GraphPad Software Inc, La Jolla, CA). Combination index (CI) values were calculated for all tested drug concentrations according to the Chou and Talalay method [33, 34] using the following equation:

where (D)_1_ and (D)_2_ represent the dose of agent 1 and 2 used in combination to induce X% growth inhibition, and (D_X_)_1_ and (D_X_)_2_ represent the dose of agent 1 and 2 required to reach X% growth inhibition when used alone. The CI theorem then provides quantitative definition for additive effects (CI = 1), synergisms (CI<1) and antagonisms (CI>1) in drug combinations.

### *In vitro* MatrigelTM assay

The Matrigel™ (BD Biosciences, North Ryde, Australia) assay was used to determine the anti-angiogenic and vascular-disrupting properties of propranolol alone or in combination with chemotherapeutic agents, as previously described [38]. Briefly, 24-well plates were coated at 4°C with 270μL of a Matrigel™ solution (1:1 dilution in cell culture medium), which was then allowed to solidify for 1 h at 37°C before cell seeding. For the anti-angiogenesis assay, endothelial cells were treated with different drug solutions 20 min after seeding on Matrigel™ and photographs were taken after 8h drug incubation using the 5X objective of an Axiovert 200M fluorescent microscope coupled to an AxioCamMR3 camera driven by the AxioVision 4.7 software (Carl Zeiss, North Ryde, Australia). For the vascular-disruption assay, endothelial cells were first allowed to undergo morphogenesis and form capillary-like structures for 6 h before drug treatment was initiated. Photographs were then taken using the same microscope device after 2h drug incubation. The anti-angiogenic and vascular-disrupting activities of the compounds were then quantitatively evaluated by measuring the total surface area of capillary tubes formed in at least 10 view fields per well using AxioVision 4.7 software. Time-lapse videomicroscopy was also employed to further evaluate the effects of propranolol on the anti-angiogenic activity of chemotherapeutic agents, as previously described [38]. Cells were constantly maintained at 37°C and 5% CO_2_ and photographs were taken every 5 min from the beginning of drug treatment and for 9 h.

$$CI=\frac{{\left(D\right)}_{1}}{{\left(D_{X}\right)}_{1}}+\frac{{\left(D\right)}_{2}}{{\left(D_{X}\right)}_{2}}$$

### Orthotopic breast cancer model

To evaluate the effect of combining propranolol with chemotherapy on tumor growth and survival, 7 weeks-old NMRI nude mice were injected into the mammary fat pad with 50,000 MDA-MB-231-luc-D3H2LN human breast cancer cells expressing firefly luciferase (Caliper Life Science, Villepinte, France) resuspended in 50 μL of a 30% Matrigel™ solution. Mice were randomized into 4 different groups 18 days after tumor cell injection following confirmation of engraftment. Treatment was initiated 3 weeks after injection and consisted of i.p injections of i) saline (5 days a week for up to 5 weeks), ii) paclitaxel alone (20 mg/kg, 3 days a week for 3 weeks), iii) propranolol alone (10 mg/kg, 5 days a week for 5 weeks) or iv) the combination of paclitaxel and propranolol following the same administration schedule. Tumor growth was monitored by weekly bioluminescence measurements using the Spectrum IVIS system (Caliper Life Science, Villepinte, France). Briefly, light expressed as photon/sec was measured 15 minutes after i.p injection of 200 mg/kg Luciferine (Caliper Life Science, Villepinte, France), a time-lag ensuring the signal to reach a plateau. After acquisition, signals were converted into 3D pictures using the Living Image 4.2 software (Caliper Life Science, Villepinte, France). Mice were sacrificed when tumor size reached 5,000 mm^3^ (as determined using a vernier caliper and the standard formula tumor volume = (thickness × length × width) × π / 6) or when signs of morbidity became apparent (e.g., ataxia, signs of pain or distress, loss of > 15% of initial weight, loss of mobility due to tumor growth).

The combination of propranolol and 5-FU was also evaluated using the same orthotopic model of triple-negative breast cancer with slight modifications. Briefly, 7 weeks-old NMRI nude mice were injected into the mammary fat pad with 10,000 MDA-MB-231-luc-D3H2LN cells in absence of Matrigel™. Mice were randomized into 4 different groups 7 days after tumor cell injection following confirmation of engraftment. Treatment was initiated 2 weeks after injection and consisted of i.p injections of i) saline (5 days a week for up to 8 weeks), ii) 5-FU alone (30 mg/kg, 3 days a week for 5 weeks), iii) propranolol alone (10 mg/kg, 5 days a week for up to 8 weeks) or iv) the combination of 5-FU and propranolol following the same administration schedule. Tumor growth was monitored by weekly bioluminescence measurement using the same protocol and device described above. Mice were sacrificed when tumor size reached 5,000 mm^3^ or when signs of morbidity became apparent.

### Statistical analysis

All *in vitro* experiments were performed at least in triplicate and statistical significance was determined using two-sided student's t test and one-way Anova was used for multiple comparisons. For *in vivo* studies, the log rank test was used to compare median survival in the different treatment groups. All statistical analyses were performed using GraphPad Prism 4 (GraphPad Software, Inc).

## Supplementary Videos and Figures

Videos 1-4: In vitro angiogenesis assay by time-lapse videomicroscopy Representative time-lapse videomicroscopy of HMEC-1 cells on Matrigel™. Cells were seeded on Matrige™ either in the absence of drug (video 1) or in the presence of 10 μM propranolol (video 2), 10 nM paclitaxel (video 3) or a combination of 10 μM propranolol and 10 nM paclitaxel (video 4). Photographs were taken every 5 min for 9 h, using the 5X objective of an Axiovert 200M fluorescent microscope coupled to an AxioCamMR3 camera driven by the AxioVision 4.7 software (Carl Zeiss). The videos together with the supplementary figures 5 & 6 can be found online at www.impactjournals.com/oncotarget










