# Supplementary material for: Propranolol potentiates the anti-angiogenic effects and anti-tumor efficacy of chemotherapy agents: implication in breast cancer treatment
Source: Oncotarget. 2011 Oct 17;2(10):797–809. doi: 10.18632/oncotarget.343 (PMC3248157; doi:10.18632/oncotarget.343)
Supplement: Supplementary file 5 [file oncotarget-02-797-s005.pdf]

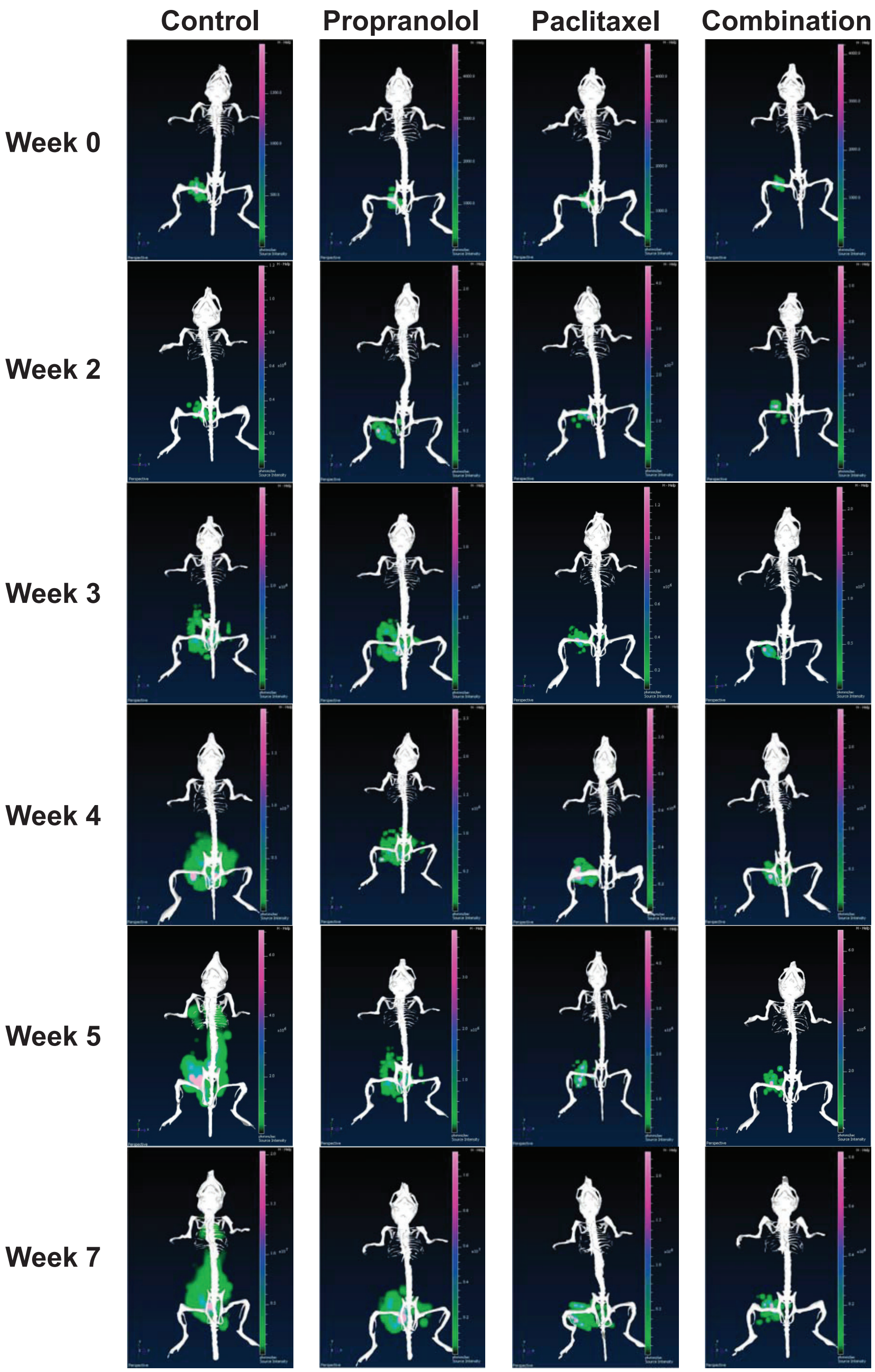

Figure 5: Illustration of 3D reconstruction of bioluminescence measurements
Representative pictures of NMRI nude mice treated with saline, propranolol alone, paclitaxel alone or the combination of propranolol and paclitaxel, starting 3 weeks after s.c injection of 50,000 MDA-MB-231-luc-D3H2LN cells into the mammary fat pad. Pictures were converted from bioluminescence measurements performed on the Spectrum IVIS system 15 minutes after i.p injection of 200 mg/kg luciferine. The color scale indicates the signal intensity, which is directly proportional to the number of tumor cells.

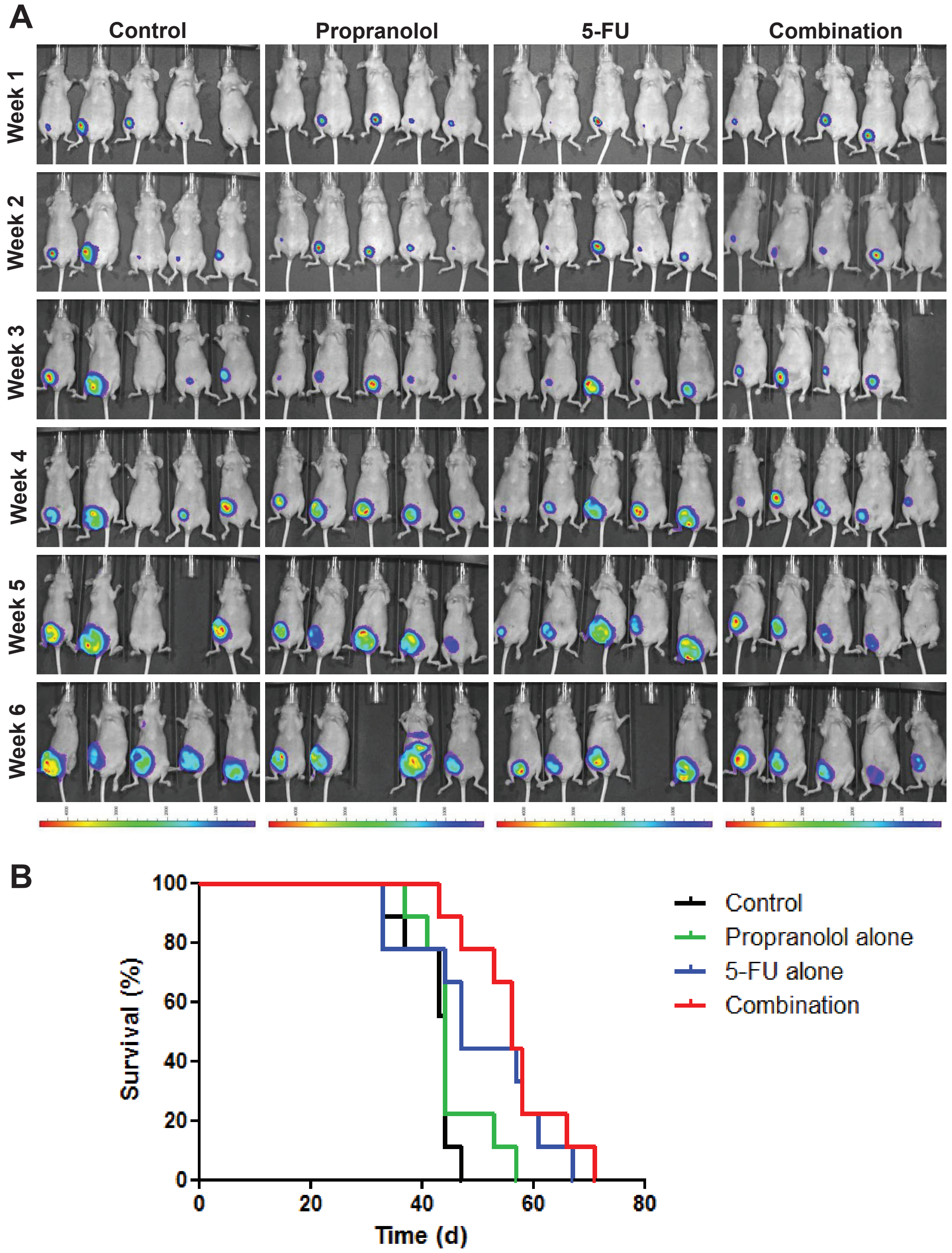

Figure 6: *In vivo* drug combination study of propranolol and 5-FU
NMRI nude mice were injected with luciferase-expressing MDA-MB-231 cells into the mammary fat pad and randomized into 4 groups (n=9 in each group) following confirmation of engraftment. Treatment was initiated 2 weeks after tumor cell injection and consisted of i.p injection of saline 5 days a week for up to 8 weeks (*black*), 10 mg/kg propranolol alone 5 days a week for up to 8 weeks (*green*), 30 mg/kg 5-fluorouracil alone 3 days a week for 5 weeks (*blue*) or the combination of propranolol and 5-fluorouracil (*red*). ***A***) Representative pictures of weekly bioluminescence measurements performed after treatment was initiated; time is expressed in weeks since start of treatment. The color scale indicates the signal intensity, which is directly proportional to the number of tumor cells. ***B***) Kaplan-Meier survival curve; time is expressed in days since start of treatment.
